# Bias-Exchange Metadynamics Simulation of Membrane Permeation of 20 Amino Acids

**DOI:** 10.3390/ijms19030885

**Published:** 2018-03-16

**Authors:** Zanxia Cao, Yunqiang Bian, Guodong Hu, Liling Zhao, Zhenzhen Kong, Yuedong Yang, Jihua Wang, Yaoqi Zhou

**Affiliations:** 1 Shandong Provincial Key Laboratory of Biophysics, Institute of Biophysics, Dezhou University, Dezhou 253023, China; qiayilai@mail.ustc.edu.cn (Z.C.); bianyuqiang@163.com (Y.B.); xzszhgd@163.com (G.H.); zhaoll@sina.com (L.Z.); kzzliweiwei@163.com (Z.K.); 2 College of Physics and Electronic Information, Dezhou University, Dezhou 253023, China; 3 College of Life Science, Shandong Normal University, Jinan 250014, China; 4 Institute for Glycomics and School of Information and Communication Technology, Griffith University, Parklands Dr, Southport, QLD 4222, Australia; yuedong.yang@griffith.edu.au; 5 School of Data and Computer Science, Sun Yat-sen University, Guangzhou 510275, China

**Keywords:** metadynamics simulation, permeation of amino acids, translocation free energy

## Abstract

Thermodynamics of the permeation of amino acids from water to lipid bilayers is an important first step for understanding the mechanism of cell-permeating peptides and the thermodynamics of membrane protein structure and stability. In this work, we employed bias-exchange metadynamics simulations to simulate the membrane permeation of all 20 amino acids from water to the center of a dipalmitoylphosphatidylcholine (DPPC) membrane (consists of 256 lipids) by using both directional and torsion angles for conformational sampling. The overall accuracy for the free energy profiles obtained is supported by significant correlation coefficients (correlation coefficient at 0.5–0.6) between our results and previous experimental or computational studies. The free energy profiles indicated that (1) polar amino acids have larger free energy barriers than nonpolar amino acids; (2) negatively charged amino acids are the most difficult to enter into the membrane; and (3) conformational transitions for many amino acids during membrane crossing is the key for reduced free energy barriers. These results represent the first set of simulated free energy profiles of membrane crossing for all 20 amino acids.

## 1. Introduction

One of the key molecular interactions in living organisms is the interaction between amino acids and lipid bilayers of cell membranes. Lipid bilayers consist of amphipathic phospholipids that are self-assembled with long hydrophobic tails buried in the center and hydrophilic heads facing the intracellular (cytosolic) and extracellular water environment. About 25% of the membrane surface is occupied by integral membrane proteins [1] that serve as gatekeepers, transporters, or signal transducers [2]. The interactions between amino acids and lipids govern the structure folding and functions of integral membrane proteins.

Cell membranes prevent the entrance of undesirable extracellular molecules so as to protect the interior of cells. However, cell membranes are permeable to some peptides. These cell-permeating peptides (CPP), which have highly different efficiency [3,4], are classified into three classes: positively charged amino acid residues, alternative pattern of polar and non-polar amino acid residues, and hydrophobic peptides with low net charge [4]. Cell-permeating peptides have been actively investigated as antibiotics [5] and cell-specific drug delivery agents [6]. However, the mechanism of cell permeation is poorly understood and such understanding requires an improved knowledge of how amino acids interact with the membrane.

One experimental strategy to probe energetics of amino acids is to measure partitioning between water and a lipid environment [7]. For example, Wimley and White measured the transfer free energy of short peptides (Ace-WLxLL) from water and a palmitoyloleoylphosphatidylcholine (POPC) membrane [8] and showed preference of aromatic but not charged residues at the membrane hydrophobic center. Hessa et al. measured a “biological hydrophobicity scale” by observing how peptides with different residues were selected for inserting into or transporting out of the membrane by translocation machinery Sec translocon [9]. More recently, McDonald and Fleming performed mutations of alanine to aromatic residues in the outer membrane protein phospholipase A (OmpLA) and demonstrated the dependence of transfer free energy of aromatic side chains on the depth inside the membrane [10]. Consistent with their partitioning coefficients, direct measurement of permeability of membranes to amino acids indicated that hydrophobic amino acids are much more permeable than hydrophilic ones [11].

Computationally, partitioning of amino acids in hydrophilic and hydrophobic environments can be estimated from protein structures according to percentages of buried residues and protein stability [12], and were found useful in locating trans-membrane helices [13]. Distributions of amino acid residues in transmembrane helical proteins revealed vastly different statistical depth-dependent profiles for different residues [14]. Molecular dynamics simulations have also played a significant role in understanding the interaction between amino acid residues and the cell membrane at atomic details. For example, experimental partitioning of 10 short peptides (Ace-WLxLL) at water/cyclohexane and water/phospholipid interfaces can be interpreted at molecular level by molecular dynamics simulations [15,16]. Distribution of 17 amino-acid analogs (without main-chain backbone atoms) across a dioleoylphosphatidylcholine (DOPC) bilayer [17] was obtained by umbrella sampling and revealed the importance of water defects in permeation of polar and charged residues. Other studies focused on the permeation mechanism of specific charged residues such as charged tryptophan [18] and arginine [19,20].

In this study, we performed bias exchange metadynamics simulations of the permeation of 20 standard amino acids from water to the center of a large Dipalmitoylphosphatidylcholine (DPPC) membrane system (consisting of 256 molecules). The large membrane system was chosen because the energy profile of a permeating amino acid is quantitatively affected by the size of the lipid bilayer [20]. DPPC was chosen because it is a commonly used model for biological membranes of mammalian cells [21]. Metadynamics molecular dynamics simulations explore free energy landscapes along several pre-chosen collective variables by using positive Gaussian potentials to bias against previously visited regions [22,23,24]. It has been successfully applied to the water-to-membrane transfer of small molecules and compared to experimental measurement [25,26,27,28]. In particular, it has successfully found that the conformational transition of aspirin [25] plays an important role for accurate estimate of transfer free energy.

To our knowledge, this is the first systematic study for membrane permeation of all 20 amino acids. Unlike the previous study of 17 side chain analogues [17], both backbone and side chain atoms of amino acids are included in our simulations to better mimic the permeation of the whole amino acid. We found that hydrophobic and positive charged amino acids are easier to penetrate into the cell than polar and negatively charged amino acids. Correlation analysis between free energy cost of permeation and physico-chemical properties of amino acids suggest hydrophobicity as the main driving force of cell permeation.

## 2. Results

### 2.1. Examination of Convergence

**Figure 1:**
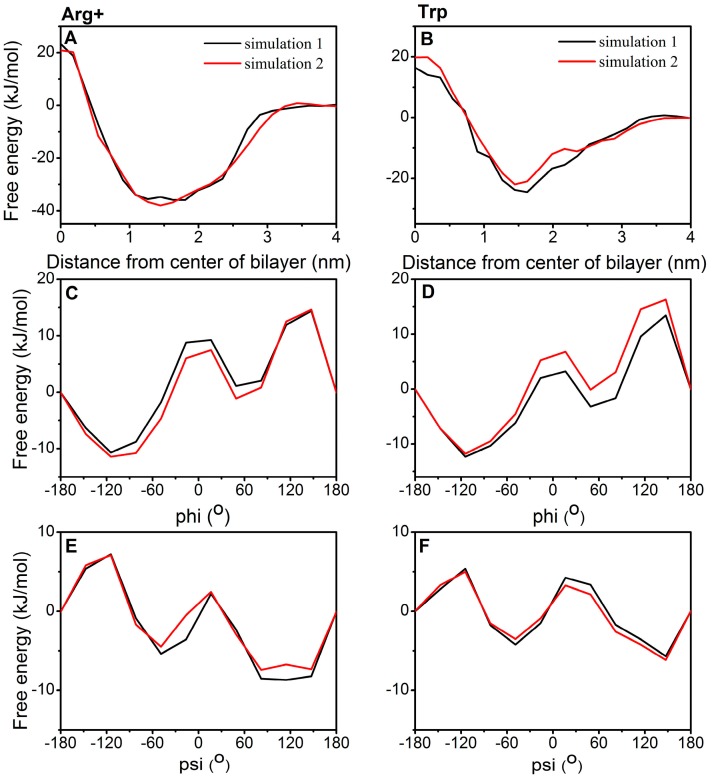
Free energy profiles (FEPs) as a function of CV1 (z-direction) (**A**,**B**), CV2 (φ angle) (**C**,**D**), and CV3 (ψ angle) (**E**,**F**) from two independent simulationsfor Arg+ (**A**,**C**,**E**, respectively) and Trp (**B**,**D**,**F**, respectively). Region I (0–0.9 nm) contains only hydrophobic lipid tails, which ranges from the center of the bilayer to the position with the highest lipid-tail density. Region II (0.9–1.4 nm) is between the position with the highest lipid-tail density and the position at the lowest phosphate density. Region III (1.4–2.2 nm) contains mostly charged phosphates. Region IV (>2.2 nm) is made of mostly water layers.

Because simulating membrane permeation of an amino acid requires long simulation time, we performed two separate calculations with different initial conformations and velocities for Arg+ and Trp to determine simulation parameters required for the overall convergence of simulations. One-dimensional free energy profiles (FEP) along the reaction coordinates were obtained by using the program sum_hills.x [29] (the command used: sum_hills.x -file -out -ndim -ndw -kt -ngrid -fix -aver). The best final estimate of the FEP is obtained by averaging the bias profiles after the filling time with the option -aver (-aver is used to plot the time average of the bias profile over the last given number of hills, see Equation (33) in ref [30]). These FEPs were shifted to be zero in the water phase or −180° for φ and ψ to facilitate the comparison. Figure 1 compared these independent FEPs of Arg+ (left panel) and Trp (right panel) as a function of z, φ, and ψ from two simulations. FEPs in the z direction as well as in φ and ψ angles were quantitatively similar to each other. For example, two independent barrier heights at the *z*-direction are 23.3 and 20.9 kJ/mol (10% difference) for Arg+, respectively, and 17.4 and 19.8 kJ/mol (12% relative difference) for Trp, respectively. Here, these two amino acids are chosen for parameter setting because one represents a positively charged residue and the other represents a residue of large size. These two residues are expected to be more difficult to converge than most other residues. 

We further examined the convergence of FEPs for 20 amino acids by comparing the profiles calculated from different simulation lengths. As shown in Figures S1–S3 (nonpolar amino acids, polar amino acids and charged amino acids, respectively), different simulation lengths yielded essentially the same free energy profiles. This convergence is due to repeated exploration of the whole sampling space as illustrated by the time evolution of CV1 (z-projection) shown in Figure S4.

### 2.2. FEPs as a Function of z for 20 Natural Amino Acids

**Figure 2:**
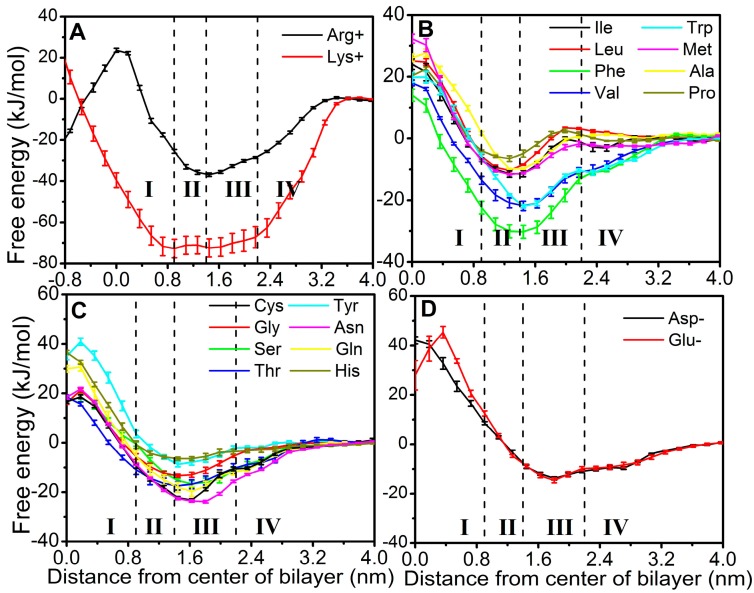
Free energy profiles (FEPs) for 20 natural amino acids as a function of the distance from the center of bilayer. (**A**) Positively charged residues; (**B**) hydrophobic residues; (**C**) hydrophilic residues, and (**D**) negatively charged residues. All FEPs are set to zero in the water phase. Region I (0–0.9 nm) contains only hydrophobic lipid tails, which ranges from the center of the bilayer to the position with the highest lipid-tail density. Region II (0.9–1.4 nm) is between the position with the highest lipid-tail density and the position at the lowest phosphate density. Region III (1.4–2.2 nm) contains mostly charged phosphates. Region IV (>2.2 nm) is made of mostly water layers.

Figure 2 compares free energy profiles (FEPs) of 20 amino acids along the z direction. The error bars were obtained based on the profiles generated from different simulation lengths as shown in Figures S1–S3. The equilibration period was excluded. These amino acids are classified into four groups: positively charged amino acid residues including Arg+ and Lys+ (Figure 2A), nonpolar amino acid residues including Ile, Leu, Phe, Val, Trp, Met, Ala and Pro (Figure 2B), polar amino acid residues including Cys, Gly, Ser, Thr, Tyr, Asn, Gln and HisA (Figure 2C), and negatively charged amino acid residues including Asp- and Glu- (Figure 2D). All PMFs have a line of symmetry through its center (z = 0) except Lys+ due to reproducible, altered conformation of one lipid (see below).

All residues can enter through the membrane from region IV (water) to region III (charged phosphate) without a significant barrier. However, only nonpolar and positively charged amino acids can reach region II (the first half of the lipid tail from phosphate) without a significant barrier. The free energies for negatively charged residues Asp- and Glu-steadily increase when moving further from region III toward the center of the membrane. All polar amino acids gain free energy in entering into the region of charged phosphate but encounter an uphill barrier when entering into the region II. Most nonpolar amino acid residues, however, can enter into the region II from bulk water in a nearly downhill fashion. This explains the fact that nonpolar amino acids are more efficient in cell permeating. Lys+ and Arg+ have two lowest free energies (−37 kJ/mol for Arg+, −72 kJ/mol for Lys+) at the interface between region II and III, indicating that Arg+ and Lys+ bind more strongly to the head group of membrane than other amino acids. The strong interaction between Lys+ and the head group of lipid bilayers leads to flipping of one lipid in the top leaflet toward the bottom leaflet. It is noted that some residues have the highest barrier located before the center (*z* = 0). This is likely due to the fact that the lipid has the lowest density at the center.

The free energy costs of 20 natural amino acids from the water to the center of the bilayer and from the minimum to the center of the bilayer are shown in Table 1. Notably, positively charged amino acids have lower barrier heights than negatively charged ones in the regions of hydrophobic tails (I-II). Moreover, the former can permeate the cell membrane as easy as uncharged amino acids because of larger free-energy gains upon entering inside the membrane (−37 kJ/mol for Arg+ and −72 kJ/mol for Lys+).

**Figure 3:**
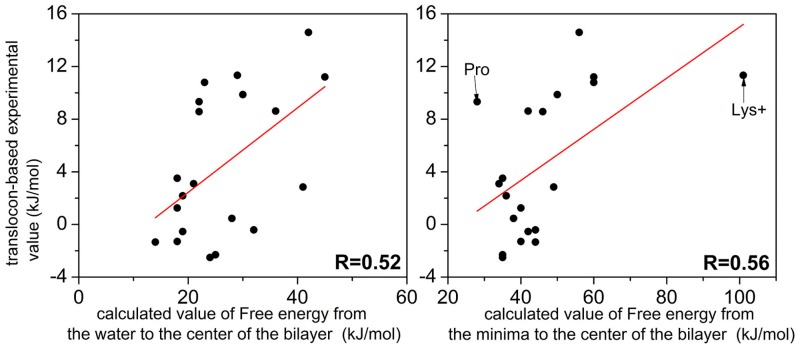
The correlation between experimental values (*y*-axis) and the calculated free energy costs (*x*-axis) of the 20 natural amino acids from the water to the center (**Left panel**) and from minimum to the center of the bilayer (**Right panel**).

The membrane-permeating propensity was indirectly measured in experiments by Hessa et al. [9], where peptides with different compositions of amino acids were selected to insert into or transporting out of the membrane by translocation machinery Sec translocon. We examined the correlation between experimental results and the simulated values (free energy costs from the water to the center of the bilayer and from the minima to the center) in Figure 3. These two sets of values are correlated with a correlation coefficient of 0.52 between experimental and simulated water-to-center free-energy cost and 0.56 between experimental and simulated minimum-to-center free-energy cost, respectively. The correlation coefficient between experimental and simulated minimum to center free energy cost increased to 0.76 if we excluded Lys+ and Pro. We also examined the correlation between calculated value of 17 amino-acid analogs (without main-chain backbone atoms) across a dioleoylphosphatidylcholine (DOPC) bilayer [17] and the simulated values (free energy costs from the water to the center of the bilayer), the correlation coefficient is 0.54. The reasonable correlation indicates consistent overall behavior, despite the difference between computational and experimental systems.

To help interpret the above free energy profiles, we calculated the correlation coefficient between the calculated free energy cost and various physico-chemical and biochemical properties of amino acids collected in the database AAindex [31,32]. There are 11 properties (Supplementary Table S1) with the absolute value of the correlation coefficient of >0.7 to either the water-to-center or minimum-to-center free energy cost of an amino acid. Majority of these properties are related to hydrophobicity and average accessible surface area. One interesting exception is high correlation (R = 0.55) between the calculated free energy cost and the STERIMOL length of the side chain (this parameter represents the length of the side chain measured in the direction in which it is attached to the glycine backbone). To locate the largest contributor to the correlation, we performed a partial least squares (PLS) regression against these properties and calculated Variable Importance in Projection (VIP) [33] that describes the importance of an independent variable for explaining variation in the dependent variable. A VIP value of 1.0 or more is a threshold for determining the importance of a variable. We found that the information value for accessibility [34] and hydropathy scale [35] has the highest VIP value (>7). The rest are all <2. The information value for accessibility is a scale of solvent accessibility of amino acid residues based on the information theory formalism. These values are positive for buried residues (mostly hydrophobic residues) and negative for exposed residues (mostly hydrophilic residues). The hydropathy scale is more positive for hydrophobic amino acid and negative for hydrophilic amino acid. These results suggest that hydrophobicity is the dominant driver for cell permeation.

### 2.3. Torsion Angle Dependence

It is of interest to know if there is orientation dependence during membrane permeation of amino acids. We calculated the free energy profiles as a function of φ and ψ, respectively, using the program sum_hills.x (command used: sum_hills.x -file -out -ndim -ndw -kt-ngrid -fix -aver). The results are shown in Supplementary Figures S5 and S6, respectively. It is clear that most residues have similar angle preference with two minima at about −90° and 60° for φ and −60° and 120° for ψ. The only two exceptions are Gly (no preference except unfavorable between −60° and 60°) and Pro (only one minimum at about −90° for φ and 150° for ψ). The most favorable regions are −180° < φ < −30° and 60° < ψ < 180°, which is in the region of a β-sheet conformation [36]. The other minimum is in the α helical region, suggesting possible conformational transitions in cell permeation.

**Figure 4:**
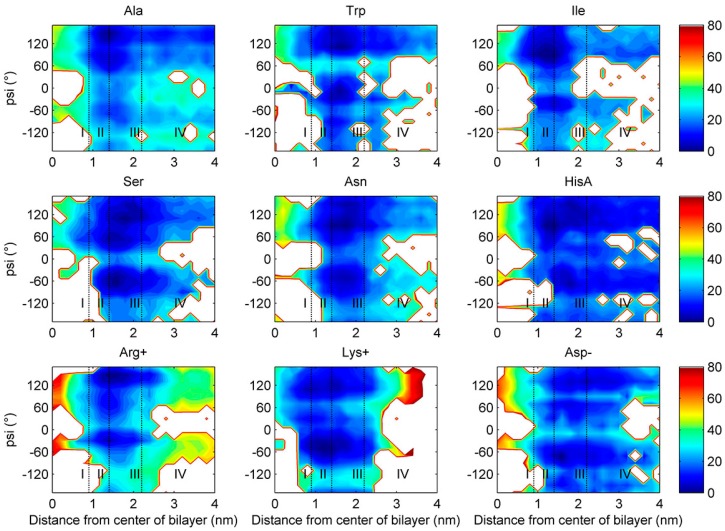
Two-dimensional free energy profiles for nine amino acids (the torsion angle ψ of the amino acid versus the distance of the amino acid from the center of lipid bilayer).

Figure 4 shows two-dimensional free energy profiles (z versus ψ) using the METAGUI3 [37] for 9 representative amino acids (the remaining shown in Supplementary Figure S7). All amino acids sampled both α helical and β-sheet regions in regions II-IV. However, at Region I (the center), many amino acids prefer β-sheet conformation only (Ile, Trp, Asn, Gln, Ser, Thr, Asp-, Glu-), a few both (Phe, Val, Gly, Tyr, Ala, Cys, HisA, Lys+, Arg+), and only one in α helical (Leu and Met). For those residues adopting one conformation at Region I, a conformation transition in backbone torsion angles from region II to region I is clearly needed for successful membrane permeation.

### 2.4. Water Defects and Pore Formation

**Figure 5:**
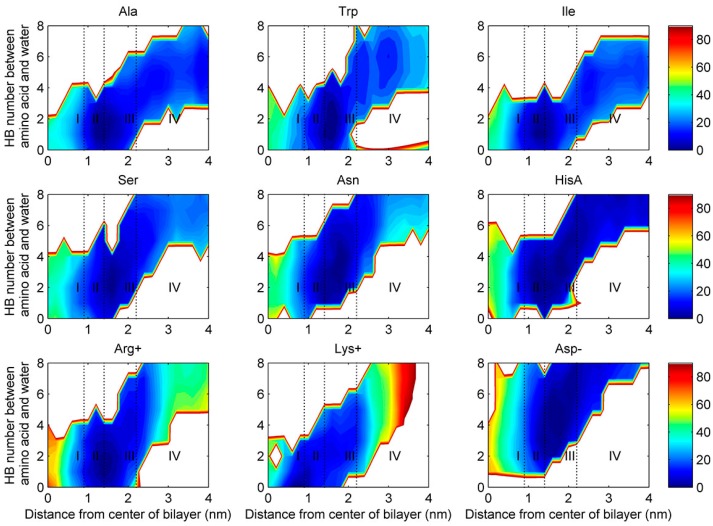
Two-dimensional free energy profiles for nine amino acids (the number of hydrogen bonds between water and the amino acid versus the distance of the amino acid from the center of lipid bilayer).

Each amino acid contains a polar main chain. Some water molecules may enter into the membrane together with the amino acid as a part of hydration shell for the main chain or the side chain (if polar) of a residue. To investigate this possibility, we calculated two-dimensional free energy landscape along the z-projection and the number of hydrogen bonds between an amino acid and water molecules by using METAGUI3. Results are shown in Figure 5 for 9 representative amino acids and the remaining in the Supplementary Figure S8. In general, polar and charged amino acids bring more water molecules with them than nonpolar amino acids. Some water molecules enter into the regions II and III with the amino acid. However, very few water molecules reached the center (region I). Thus, only a small water defect is formed when an amino acid is in the bilayer region I.

**Figure 6:**
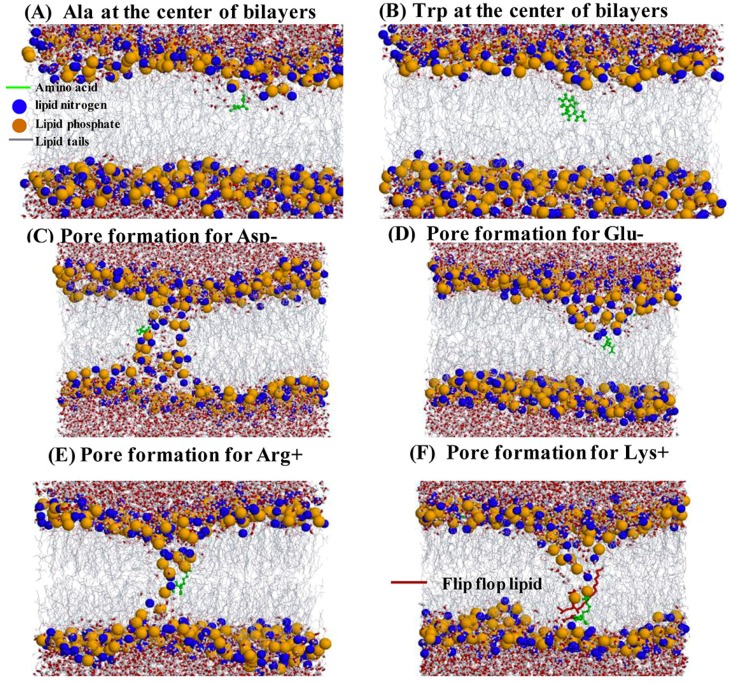
(**A**) The snapshot for Ala at the center of the bilayer where only water defect is visible. The lipid nitrogen and phosphate atoms are shown as blue and orange spheres, respectively. The lipid tails are shown as thin gray lines. Water is shown as red (oxygen) and white (hydrogen) cylinders; (**B**) The snapshot for Trp at the center of the bilayer where only water defect is visible; (**C**) Pore formation for Asp-; (**D**) Pore formation for Glu-; (**E**) Pore formation for Arg+; (**F**) Pore formation for Lys+, one lipid from the top leaflet flips flop toward the bottom leaflet as Lys+ further moved to the bottom leaflet. The lipid is shown as red.

We also evaluated the pore formation by a clustering method [38]. This method only considered the distance between phosphate atoms in the top leaflet and those in the bottom leaflet. Two phosphate atoms are considered to be in the same cluster if the distance between them is less than 1.2 nm, and thus the existence of a phosphate-atom cluster indicates the presence of a pore. We found that pore formation occurs only when Arg+, Lys+, Asp- and Glu- are at the bilayer center (shown in Figure 6). Lys+, in particular, leads to flipping of one lipid in the top leaflet toward the bottom leaflet. This disruption of membrane is consistent with the steady increase in free energy after passing through the center (Figure 2).

## 3. Discussion

In this study, we have obtained the free energy profiles of all 20 natural amino acids. In general, polar amino acids have larger free energy barriers than nonpolar amino acids, and negatively charged amino acids are the most difficult to enter into the membrane. The results are consistent with previous experimental and computational studies. The obtained highly reproducible free energy profiles benefit from the use of both directional and torsion angles for conformational sampling. We observed conformational transitions for many amino acids from helical to sheet or sheet to helical regions in backbone torsion angles during cell permeating (Figure 3 and Figure S7).

The free energy cost of the ACE-Arg+-NH2 translocation from water to the center of 256 DPPC bilayer is 23 kJ/mol. This result is lower than 60.5 kJ/mol, the free energy of transfer of cationic arginine side chain into 64 DOPC bilayer [17] and 74.4 kJ/mol into 72 DPPC bilayer [21]. Our result is much closer to 29 kJ/mol, the free energy of transfer of ACE-Arg+-NH2 into 288 DMPC bilayer [20]. However, the FES profiles are different. The FESs show a minimum of −37 kJ/mol for 256 DPPC bilayer at the interface between region II and III and −3.5 kJ/mol for 288 DMPC bilayer in the region of lipid headgroup. We also obtained the free energy cost of the ACE-Trp-NH2 translocation at about 18 kJ/mol, close to 20.9 kJ/mol, the free energy transfer of uncharged Trp into 40 DOPC bilayer [18] with similar FES curves.

Experimentally, Chakrabarti [11] summarized the order of amino acid permeability by different experiments to establish the order of amino acid permeability as Phe > Met > Leu > Ile; Leu > Ala; Gly > His. This is largely consistent with Phe > (Ile > Leu) > Met; Leu > Ala; Gly > His from our calculation on the free energy changes from water to the center. Naoi et al. [39] obtained amino acid permeability according to an order by Leu > Phe > Trp > Met > Tyr and Val > Thr >Ser > Ala > Gly. This is largely consistent with Phe > Trp > Leu > Met > Tyr and Val > Ser~Thr > Gly > Ala from our calculation. In general, the order of Leu, Phe and Met is different in different experiments [40]. The observed minor differences are likely due to the approximate nature of the membrane model system and its large difference from experimental membrane systems and experimental conditions.

To understand the mechanism of cell permeating, we performed the correlation analysis between calculated free energy profiles and physio-chemical properties of amino acids. We found that hydrophobicity plays the most important role in cell permeating. This indicates that for an non-natural amino acid, its cell permeability can be estimated from its measurable hydrophobicity. Another correlated scale is the STERIMOL length of the side chain. This suggests the intrinsic role of side chains in cell permutation in addition to hydrophobility.

## 4. Materials and Methods

**Figure 7:**
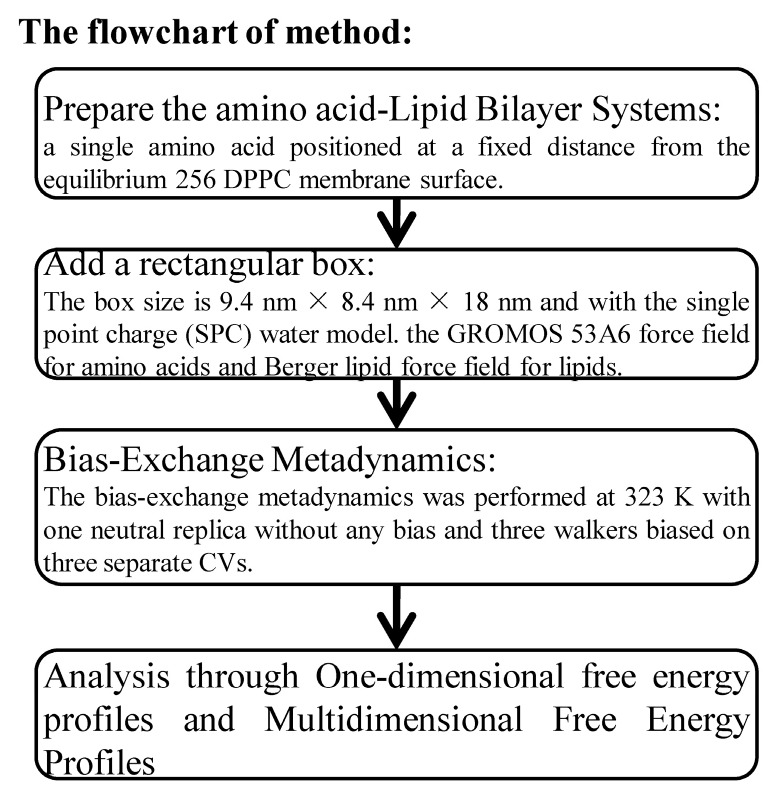
The flowchart of method.

The overall flow of the methods employed is shown in Figure 7. This includes preparation of lipid bilayer systems, solvation in a periodic boundary condition (rectangular box), simulations with bias-exchange metadyanmics, and free energy analysis. The details are described below. 

### 4.1. MD Simulation

The initial lipid bilayer system of zwitterionic DPPC was obtained from the webpage http://wcm.ucalgary.ca/tieleman/downloads. It consists of 128 lipids in each of the top and bottom leaflets (a total of 2 × 128 = 256 lipids). The final lipid bilayer system was produced after extensive minimization and 100 ns MD simulation by using the united atom force field of Berger et al. [41] and the GROMACS 4.6.2 software package (downloadable from http://www.gromacs.org/) [42]. The long-range electrostatic interaction was treated by the Particle-Mesh Ewald (PME) method with a grid spacing of 0.12 and a fourth order interpolation [43]. The distance for the coulomb cut-off is 1.2 nm. Van der Waals (VDW) interactions were calculated using a cutoff of 1.2 nm. The time step for the MD integrator was set to 2 fs with all bond lengths constrained by LINCS. All MD simulations were performed using periodic boundary conditions in a rectangular box. The box size is 9.4 nm × 8.4 nm × 18 nm.

All subsequent MD simulations employed the GROMOS 53A6 force field with the single point charge (SPC) water model [44]. We employed the GROMOS 53A6 force field for amino acids and Berger lipid force field for lipids as this combination was extensively used for studies of cell penetrating peptides interacting with zwitterionic lipid bilayers [45,46,47]. Preliminary equilibration after extensive energy minimization was performed for 2 ns in the NVT ensemble and 5 ns in the NPT ensemble. Lipid and water molecules were coupled to a heat bath at a temperature of 323 K, using a velocity rescaling with a stochastic term and a temperature coupling time of 0.1 ps. This temperature is slightly higher than the DPPC phase transition temperature (315 K) [48] to ensure the dynamics of lipid bilayers. The pressure of the system was controlled by using a semi-isotropic Parrinello−Rahman barostat with a time constant of coupling at 1 ps and a compressibility of 4.5 × 10^−5^ bar^−1^ [49]. The pressures in the direction normal to the bilayer surface and in the membrane plane were independently coupled at 1 bar.

### 4.2. Amino Acid-Lipid Bilayer Systems

**Figure 8:**
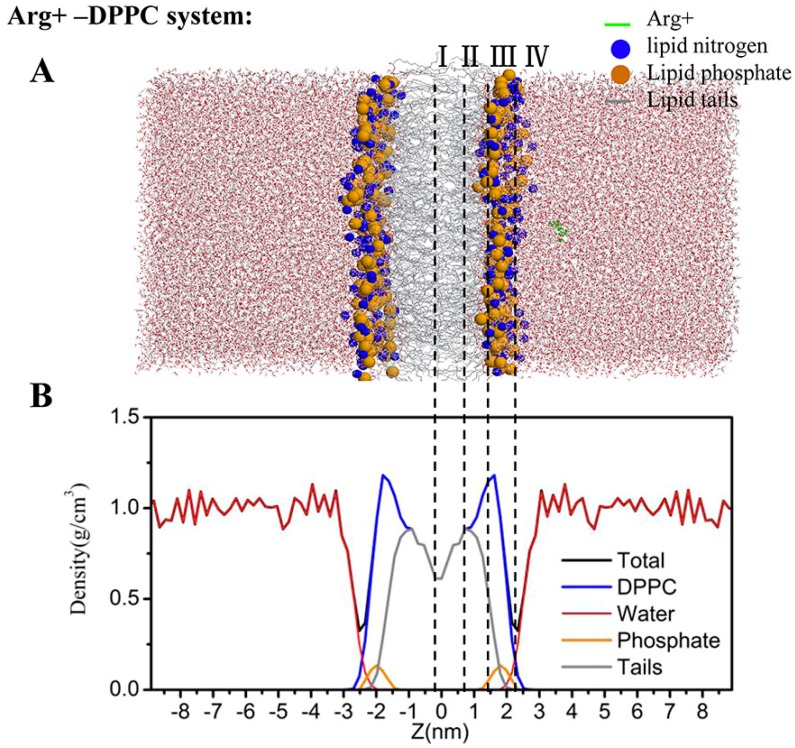
The initial simulation system (**A**) and its associated the density profiles (**B**) for the charged Arginine. In the snapshot (**A**), the amino acid is shown in green. The lipid nitrogen and phosphate atoms are shown as blue and orange spheres, respectively. The lipid tails are shown as thin gray lines. Water is shown as red (oxygen) and white (hydrogen) cylinders. The lines and roman numerals divide the system into four regions as described in the text. The density profiles of different molecules (or atoms) are shown as labeled in Figure 8B.

All amino acids were neutralized by patching with standard NH2 (CT2) group at the C-terminus, and acetyl (ACE) group at the N-terminus. Each simulation system contained a total of ~113,000 atoms, including ~33,600 water molecules, 256 DPPC molecules, and a single amino acid positioned at a fixed distance (about 1.4 nm) from the membrane surface. An example is shown in Figure 8A for charged arginine (Arg+). We added one chloride anion to neutralize Arg+ and Lys+ and one sodium cation to neutralize Asp- and Glu-. The neutral N1-H tautomer was used for His (HisA).

### 4.3. Bias-Exchange Metadynamics

All bias-exchange metadynamics (BEMD) simulations [50] were performed with GROMACS 4.6.2 [42] and PLUMED version 1.3 plugin [29]. Translocation of an amino acid through the DPPC membrane was modeled by employing three collective variables (CV): the backbone torsion angles φ and ψ of an amino acid and the z projection of the position vector between the center of mass of the amino acid and the center of mass of whole leaflet DPPC molecules. The bias-exchange metadynamics was performed at 323 K with one neutral replica without any bias and three walkers biased on three separate CVs. During the BEMD simulation, the conformations and velocities of different replicas were exchanged periodically according to a Metropolis criterion [51]. The Gaussian potentials were applied with a rate of 0.04 kJ/mol per picosecond and their widths were set to 0.2 nm, 0.314 rad and 0.314 rad, for z, φ and ψ, respectively. The attempting frequency for replica exchanges was set to 30 ps. For charged amino acids (Asp-, Glu-, Arg+, Lys+), the overall simulation time of the metadynamics was 0.8 microseconds with each replica lasting for 200 ns. For the other 16 amino acids, the overall simulation time of the metadynamics was 0.48 microseconds with each replica lasting for 120 ns. These parameters were set after examining convergence of the calculation for the free energy profiles of Arg+ and Trp by two separate simulations (Figure 1).

### 4.4. Multidimensional Free Energy Profiles

BEMD simulations allow the free energy of a system to be reconstructed after an equilibration time [50]. In our study, we have chosen the CV1 (the z projection of the position vector between the center of mass of the amino acid and the center of mass of whole leaflet DPPC molecules), CV3 (ψ) and a new CV computed at run-time (the number of hydrogen bonds between an amino acid and water molecules). All the multidimensional free energy landscapes (Figure 3 and Figure S7 as a function of z versus ψ and Figure 4 and Figure S8 as a function of z versus the number of hydrogen bonds between an amino acid and water molecules) have been performed using METAGUI3 [37] program (downloadable from https://github.com/metagui/metagui3), a visual Molecular Dynamics (VMD) [52] interface for analyzing bias-exchange metadynamics simulations.

### 4.5. Regions of Bilayer Simulations

To describe the potential of mean forces, we employed the same definition of four regions along the *z*-axis (Figure 7B), as defined in MacCallum et al. [17]. Region I (0–0.9 nm) contains only hydrophobic lipid tails, which ranges from the center of the bilayer to the position with the highest lipid-tail density. Region II (0.9–1.4 nm) is between the position with the highest lipid-tail density and the position at the lowest phosphate density. Region III (1.4–2.2 nm) contains mostly charged phosphates. Region IV (>2.2 nm) is made of mostly water layers.

## Figures and Tables

**Table 1:** The free energy costs of 20 natural amino acids from water to the center of the bilayer and from the free energy minimum to the center of the bilayer (in kJ/mol), ordered in the sequence of hydrophobic, polar, positively charged and negatively charged amino acids.

	From Water	From Minimum	Expt. (Hessa et al.)
ILE	24	35	−2.5
LEU	25	35	−2.3
PHE	14	44	−1.3
VAL	18	40	−1.3
TRP	18	40	1.3
ALA	28	38	0.5
MET	32	44	−0.4
PRO	22	28	9.3
CYS	19	42	−0.5
GLY	21	34	3.1
THR	19	36	2.2
TYR	41	49	2.8
HISA	36	42	8.6
SER	18	35	3.5
ASN	22	46	8.6
GLN	30	50	9.9
ARG+	23	60	10.8
LYS+	29	101	11.3
GLU-	45	60	11.2
ASP-	42	56	14.6

The last column is the membrane-permeating propensity which indirectly measured in experiments by Hessa et al.

## Supplementary Materials

Supplementary materials can be found at http://www.mdpi.com/1422-0067/19/3/885/s1.
