# Supplementary material for: Bias-Exchange Metadynamics Simulation of Membrane Permeation of 20 Amino Acids
Source: Int J Mol Sci. 2018 Mar 16;19(3):885. doi: 10.3390/ijms19030885 (PMC5877746; doi:10.3390/ijms19030885)
Supplement: Supplementary file 1 [file ijms-19-00885-s001.pdf]

International Journal of *Molecular Sciences*

MDPI

Supplementary Materials

Bias-Exchange Metadynamics Simulation of Membrane Permeation of 20 Amino Acids

Zanxia Cao [1,2], Yunqiang Bian [1], Guodong Hu [1,2], Liling Zhao [1,2], Zhenzhen Kong [1,3], Yuedong Yang [4,5], Jihua Wang [1,2,*] and Yaoqi Zhou [1,4,*]

1 Shandong Provincial Key Laboratory of Biophysics, Institute of Biophysics, Dezhou University, Dezhou 253023, China; qiayilai@mail.ustc.edu.cn (Z.C.); bianyuqiang@163.com (Y.B.); xzszhgd@163.com e(G.H.); zhaoll@sina.com (L.Z.); kzzliweiwei@163.com (Z.K.)

2 College of Physics and Electronic Information, Dezhou University, Dezhou 253023, China

3 College of Life Science, Shandong Normal University, Jinan 250014, China

4 Institute for Glycomics and School of Information and Communication Technology, Griffith University, Parklands Dr, Southport, QLD 4222, Australia; yuedong.yang@griffith.edu.au (Y.Y); yaoqi.zhou@griffith.edu.au (Y.Z.)

5 School of Data and Computer Science, Sun Yat-sen University, Guangzhou 510275, China

* Correspondence: jhw25336@126.com (J.W.); yaoqi.zhou@griffith.edu.au, Tel.: +61-755-528228 (Y.Z.);

Table S1. The correlation coefficients of various properties of amino acids from AAindex to the free energy costs from water to the center of the bilayer (R1) and to the free energy costs from the free-energy minimum to the center of the bilayer (R2). Only properties with either R1 or R2>0.7 are shown.

The variables from AAindex	R1	R2
Residue accessible surface area in folded protein (Chothia, 1976)_34	0.31	0.72
Hydration number (Hopfinger, 1971), Cited by Charton-Charton (1982)_114	0.68	0.70
Average accessible surface area (Janin et al., 1978)_127	0.42	0.78
Percentage of exposed residues (Janin et al., 1978)_129	0.45	0.75
Transfer free energy (Janin, 1979)_131	-0.40	-0.76
Average non-bonded energy per atom (Oobatake-Ooi, 1977)_213	0.45	0.71
Hydrophobicity (Prabhakaran, 1990)_252	0.50	0.71
Energy transfer from out to in(95%buried) (Radzicka-Wolfenden, 1988)_320	-0.30	-0.70
Average interactions per side chain atom (Warme-Morgan, 1978)_382	-0.38	-0.70
Apparent partition energies calculated from Janin index (Guy, 1985)_522	0.40	0.77
Hydrophobicity index (Engelman et al., 1986)_543	0.50	0.71

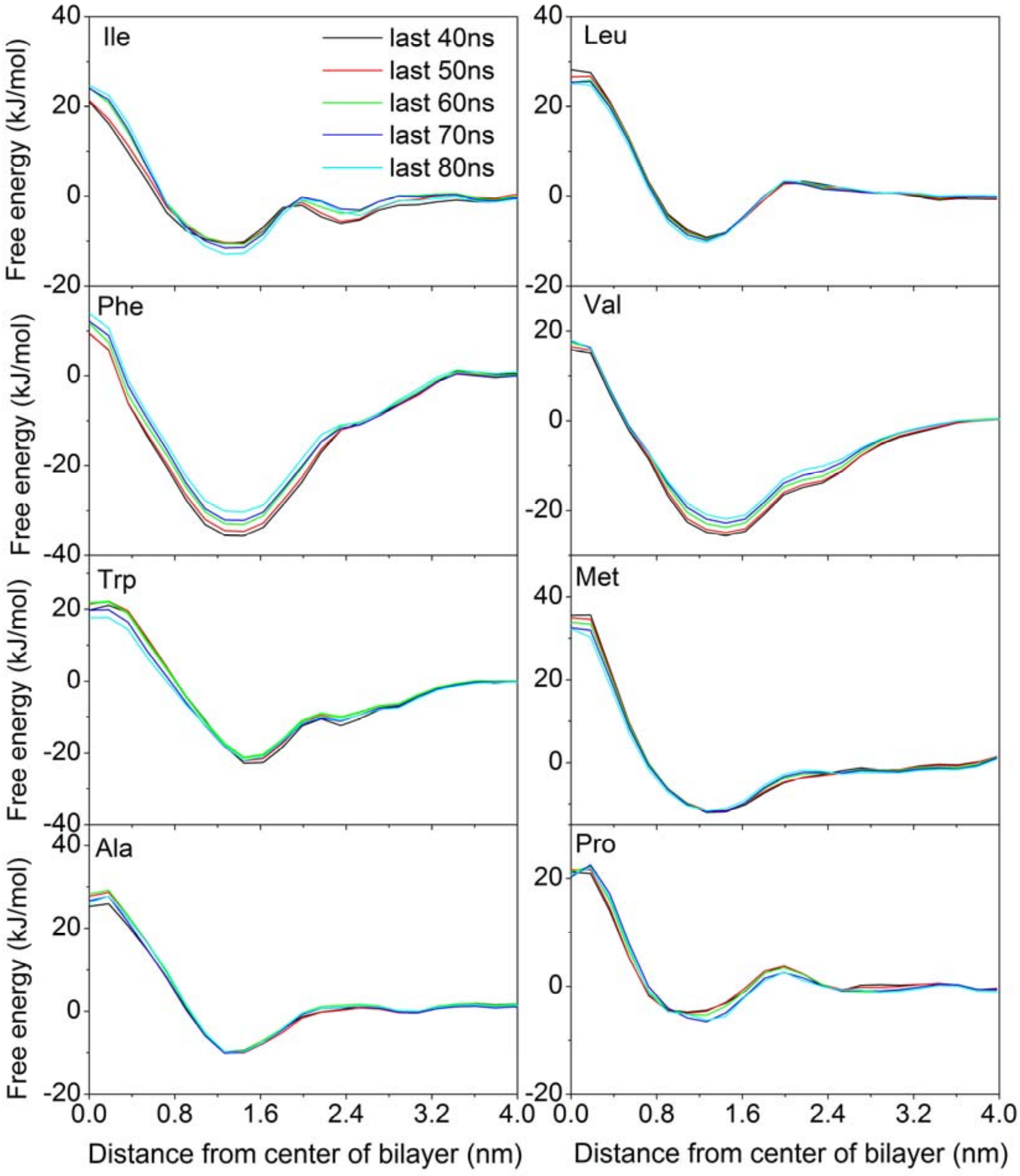

Figure S1. Free energy profiles (FEPs) for eight nonpolar amino acids (Ile, Leu, Phe, Val, Trp, Met, Ala and Pro) as a function of CV1 (the z direction) calculated by using different simulation lengths as labeled. All FEPs are set to zero in the water phase.

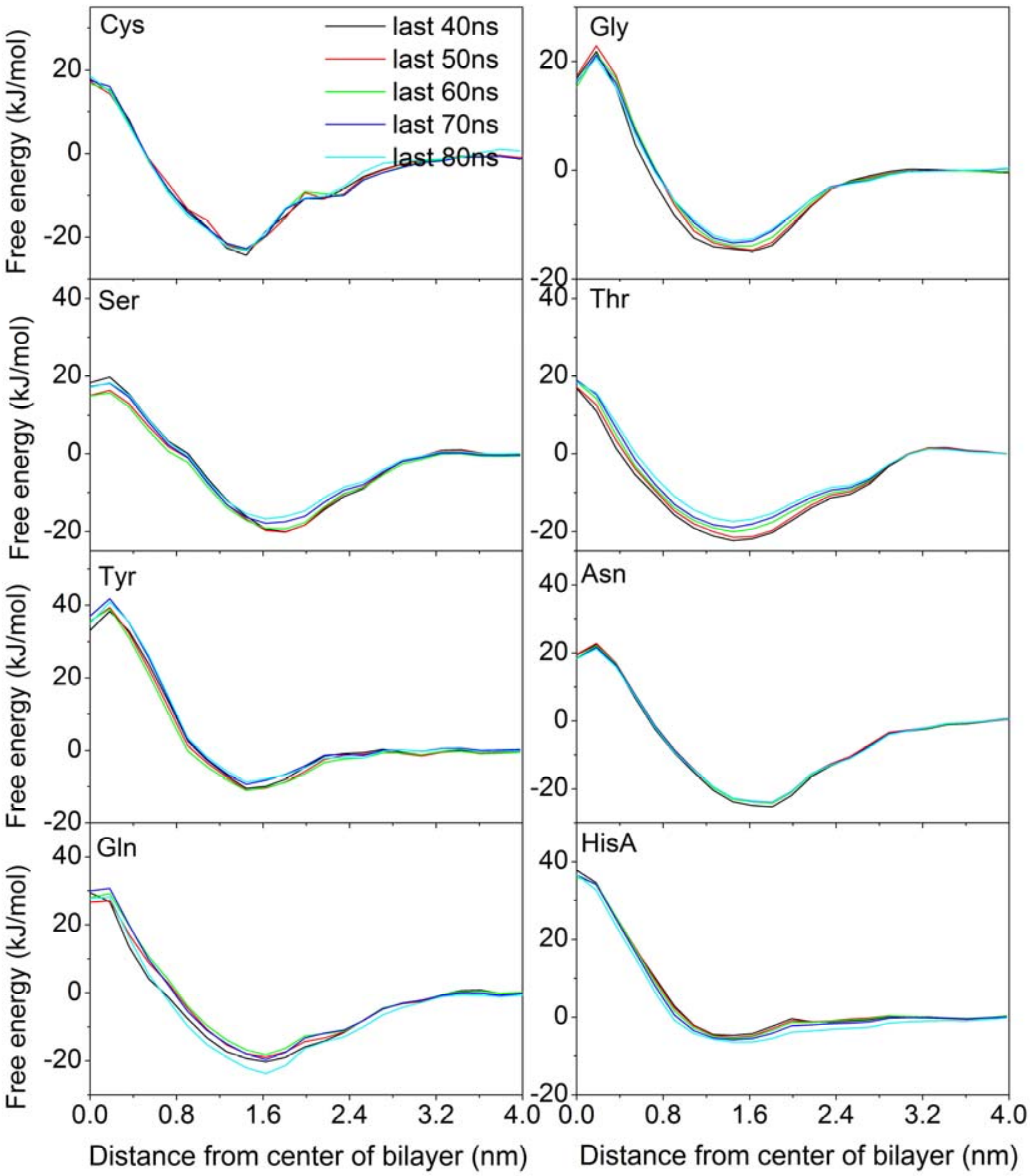

Figure S2. Free energy profiles (FEPs) for eight polar amino acids (Cys, Gly, Ser, Thr, Tyr, Asn, Gln and HisA) as a function of CV1 (the z direction) calculated by using different simulation lengths as labeled. All FEPs are set to zero in the water phase.

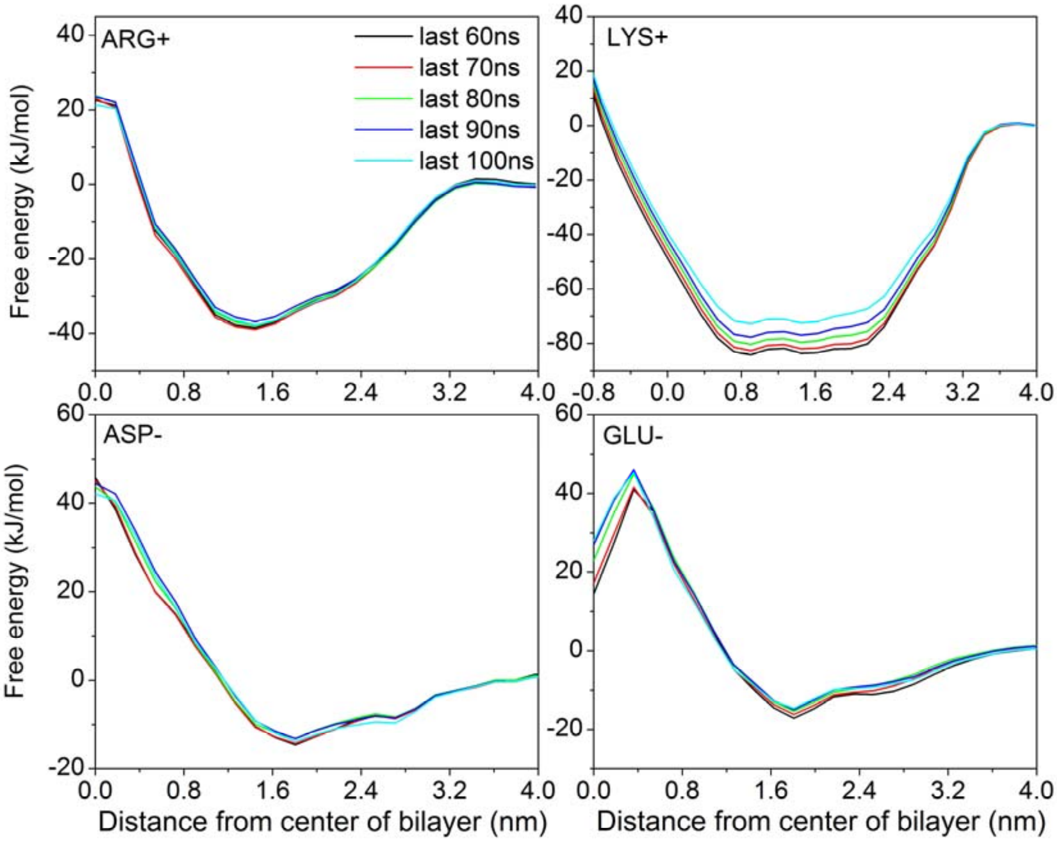

Figure S3. Free energy profiles (FEPs) for Arg+, Lys+, Asp- and Glu- as a function of CV1 (the z direction) calculated by using different simulation lengths as labeled. All FEPs are set to zero in the water phase.

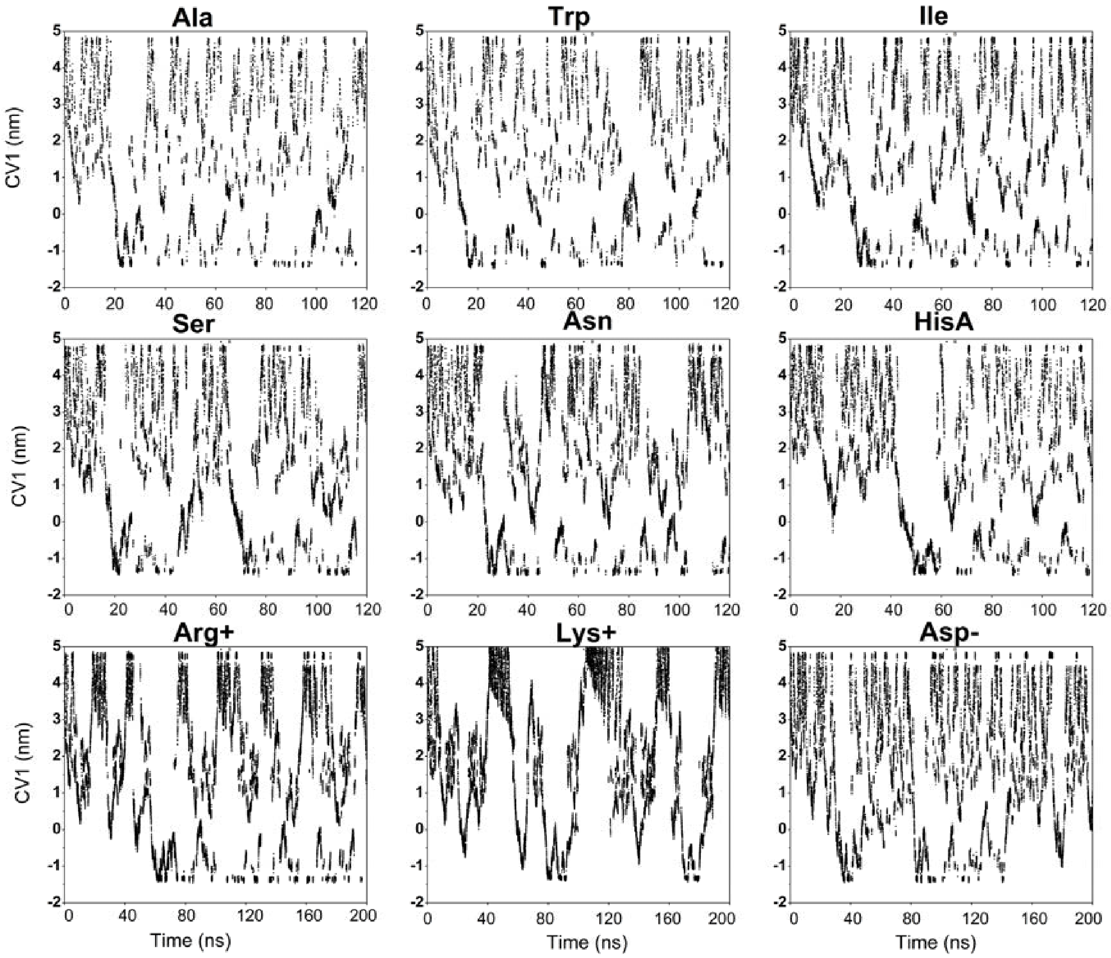

Figure S4. Time evolution of CV1 (z-projection) for nine amino acids.

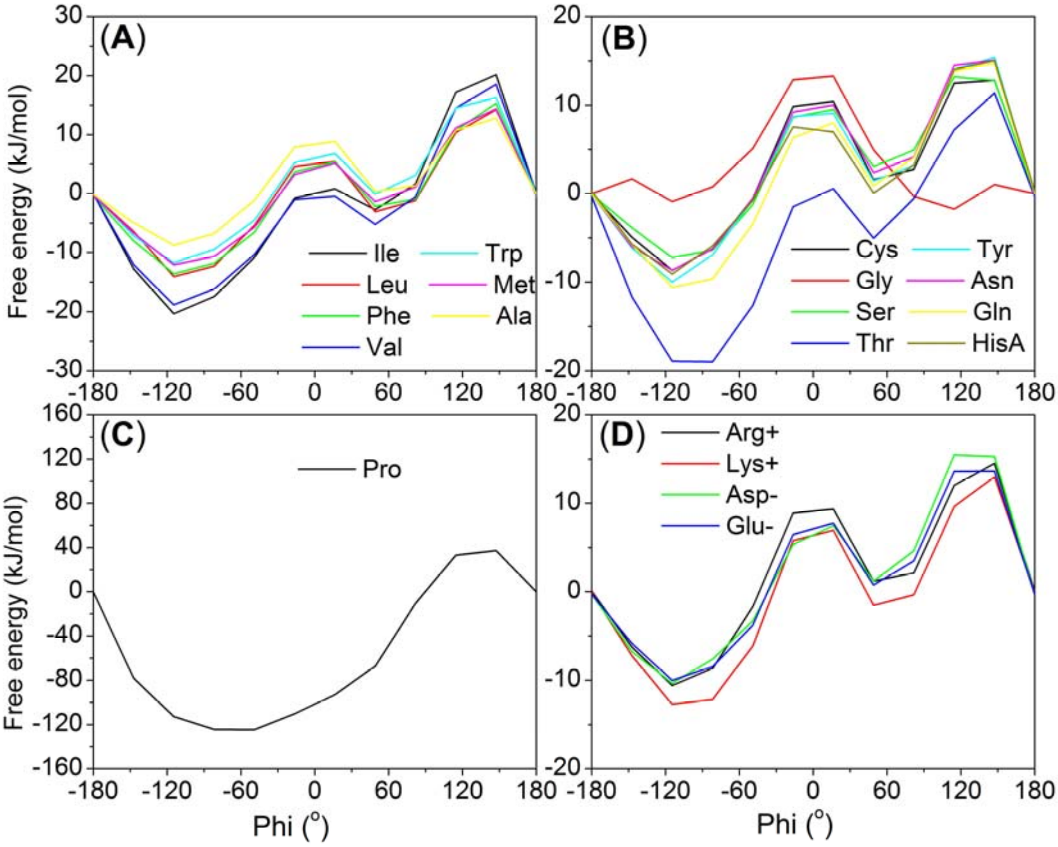

Figure S5. Free energy profiles (FEPs) for 20 natural amino acids as a function of CV2 (the backbone φ angle). All FEPs are set to zero at -180°.

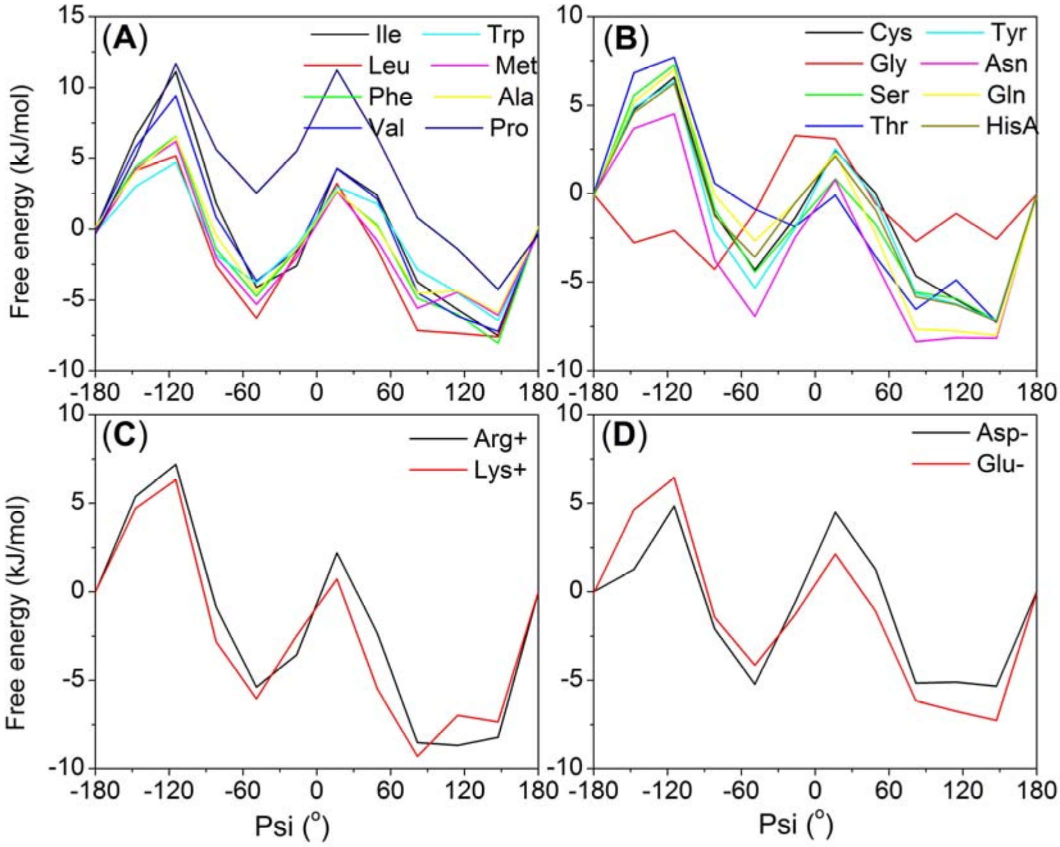

Figure S6. Free energy profiles (FEPs) for 20 natural amino acids as a function of CV3 (the backbone ψ angle). All FEPs are set to zero at -180°.

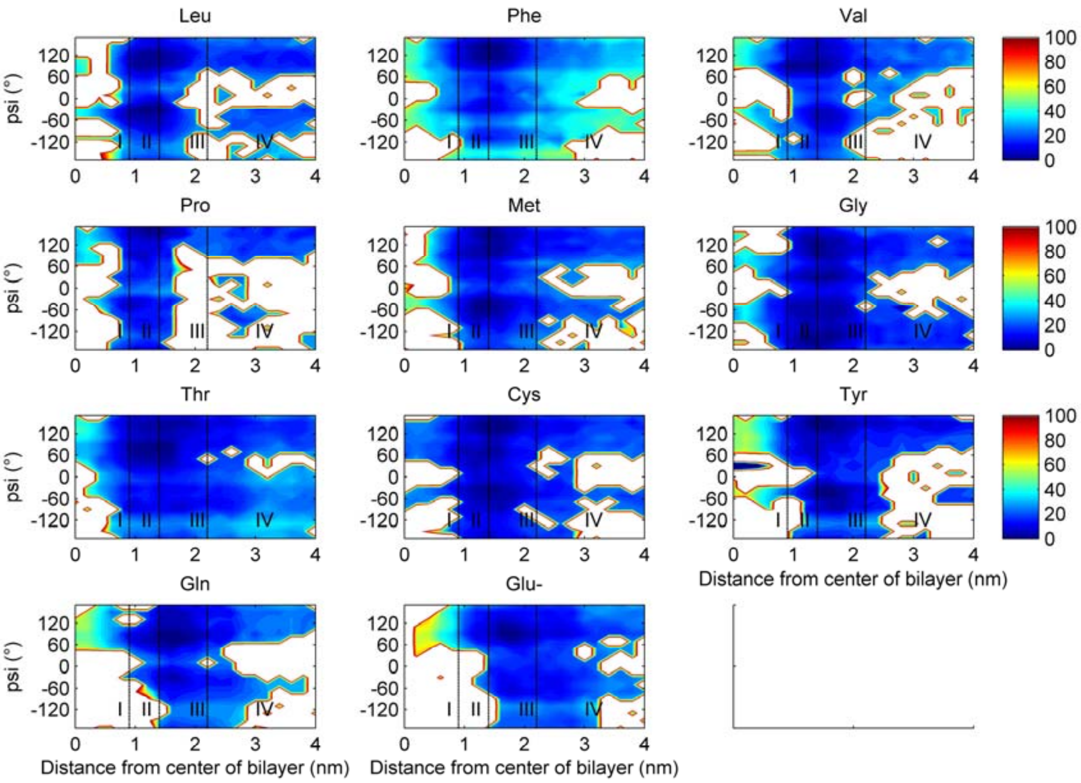

Figure S7. Two-dimensional free energy profiles (z-projection to the center versus torsion angle ψ) for eleven amino acids as labeled.

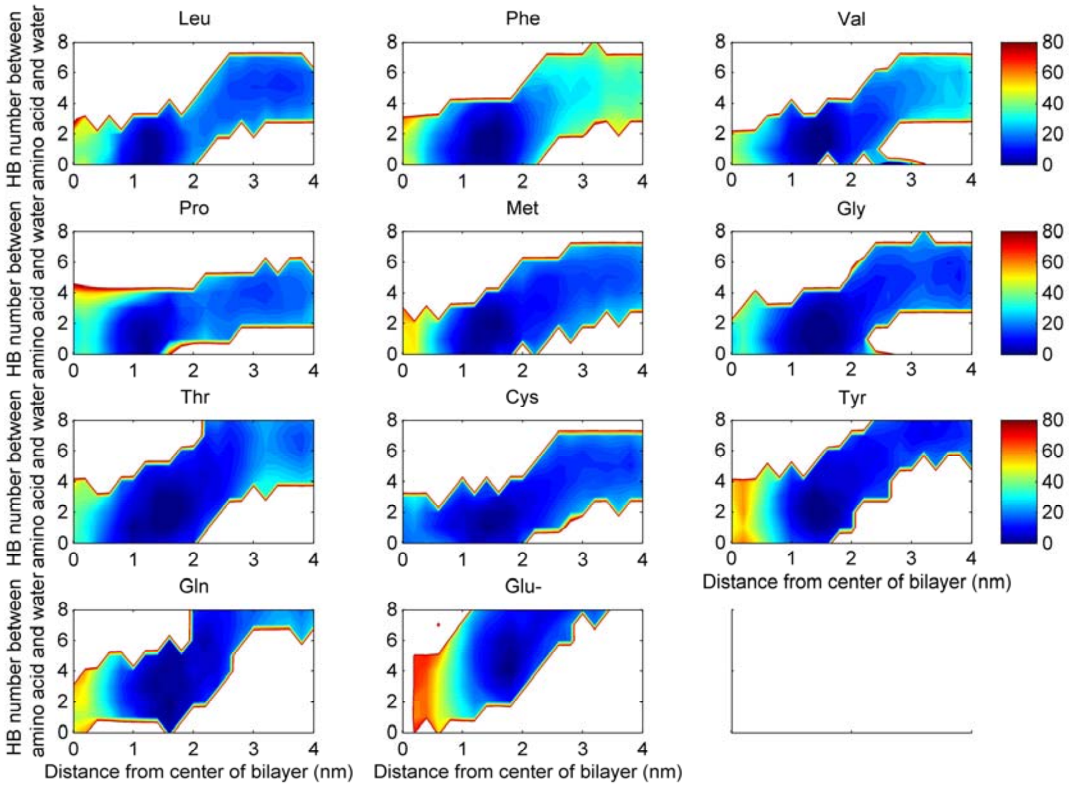

Figure S8. Two-dimensional free energy profiles (z-projection to the center versus the number of hydrogen bonds between water and the amino acid) for eleven amino acids as labeled.

© 2018 by the authors. Submitted for possible open access publication under the terms and conditions of the Creative Commons Attribution (CC BY) license (http://creativecommons.org/licenses/by/4.0/).
